# Resveratrol Treatment Induces Mito-miRNome Modification in Follicular Fluid from Aged Women with a Poor Prognosis for In Vitro Fertilization Cycles

**DOI:** 10.3390/antiox11051019

**Published:** 2022-05-21

**Authors:** Rosalia Battaglia, Angela Caponnetto, Anna Maria Caringella, Anna Cortone, Carmen Ferrara, Salvatore Smirni, Rossana Iannitti, Michele Purrello, Giuseppe D’Amato, Bernard Fioretti, Cinzia Di Pietro

**Affiliations:** 1 Department of Biomedical and Biotechnological Sciences, Section of Biology and Genetics “Giovanni Sichel”, University of Catania, 95123 Catania, CT, Italy; rosalia.battaglia@unict.it (R.B.); angela.caponnetto@unict.it (A.C.); carmen.ferrara@phd.unict.it (C.F.); salvatore.smirni@gmail.com (S.S.); purrello@unict.it (M.P.); 2 Asl Bari, Reproductive and IVF Unit, PTA “F Jaia”, 70014 Conversano, BA, Italy; a.caringella@libero.it (A.M.C.); cortone.anna@gmail.com (A.C.); g.damato@iol.it (G.D.); 3 S&R Farmaceutici S.p.A, 06083 Bastia Umbra, PG, Italy; r.iannitti@srfarmaceutici.com; 4 Department of Chemistry, Biology and Biotechnologies, University of Perugia, 06123 Perugia, PG, Italy; bernard.fioretti@unipg.it

**Keywords:** reproductive aging, poor prognosis women, Resveratrol, regulation of gene expression, microRNAs, mitochondria

## Abstract

Advanced maternal age impairs reproductive performance, influencing the quantity and the quality of oocytes. Mitochondria dysfunction seems to play a decisive role in conditioning the quality of the female gamete. Different in vitro and in vivo studies, demonstrated the antioxidant and anti-inflammatory activities of Resveratrol and its ability to improve mitochondria function even if the exact mechanism of action has not yet been demonstrated in human oocytes. In this paper, by retrospective analysis, we evaluated follicular fluid (FF) miRNome modification in aged women with a poor ovarian reserve receiving a resveratrol-based supplement the three months before the in vitro Fertilization (IVF) cycle. We found 13 differentially expressed microRNAs (miRNAs) in women treated with resveratrol and specifically miR-125b-5p, miR-132-3p, miR-19a-3p, miR-30a-5p and miR-660-5p, regulating mitochondrial proteins, are able to control metabolism and mitochondrial biogenesis. MiRNA expression differences, observed after resveratrol treatment in FF from women with a poor prognosis for IVF, demonstrated that resveratrol may act on mitomiRNAs to improve follicular microenvironment by transcriptomic and proteomic modifications in granulosa cells.

## 1. Introduction

Reproductive aging is a complex biological phenomenon concerning physiological, genetic and molecular changes [1] which begins during the fourth decade of a woman’s life, leading to increased infertility and pregnancy risks [2]. Decreasing fertility is primarily due to waves of oocyte atresia whereby growing follicles are continuously induced to undergo cell death with consequent depleting of the non-renewable ovarian reserve established during fetal development [3]. A broad range of progressive changes also occurs in oocytes thus limiting reproductive success. Different alterations in cytoplasmic and nuclear maturation processes have been described and the increase in non-disjunction meiotic errors probably represents one of the most important [4,5]. Mitochondria are the oocyte powerhouse, and through oxidative phosphorylation provide the energy for transcription and translation during oocyte maturation, fertilization, and embryonic development. In reproductive aging, several mitochondria dysfunctions, such as a decrease in their number, mtDNA damage and membrane potential instability, have been described [6]. Mitochondrial dysfunction, causing ATP deficiency with increased oxidative stress, can contribute to impairments in meiotic spindle assembly, cell cycle regulation, chromosome segregation, embryo development, and implantation [7]. Additional treatments with micronutrients, before IVF cycles, have been demonstrated to protect the follicular microenvironment from oxidative stress, increasing the number of good quality oocytes recovered at the pickup [8]. Thus, finding new strategies aiming at enhancing mitochondrial function to improve oocyte quality and age-related infertility has become the goal of a plethora of studies [6].

Different papers demonstrated that Resveratrol (3,5,4′-trihydroxystilbene), a stilbenic structure polyphenol, that under normal intracellular redox conditions, behaves as a natural antioxidant at low concentrations [9], improves mitochondria function with an induction of genes for oxidative phosphorylation and mitochondrial biogenesis. High-dose resveratrol, instead, may prompt pro-oxidant effects, inducing systemic inhibition of P450 cytochromes and mitochondrial-dependent cell death [9,10,11].

Moreover, Resveratrol, inducing SIRT1, decreases the Peroxisome proliferator-activated receptor-gamma coactivator-1alpha (PGC-1α) acetylation and increases its activity [12,13]. Its antioxidant and anti-inflammatory activities have been shown in different cellular models including human granulosa cells which enhanced metabolic activities, mitochondrial biogenesis and the global electric potential production [14]. In a recent review, the authors discuss the use of resveratrol, and other antioxidant treatments to improve human oocyte and embryo quality, focusing on the mitochondria as their main targets. However, they conclude that the mechanism of action of the treatments has not yet been demonstrated in the human oocyte and highlight the need for further studies in this field [15].

In recent years, different papers have established that polyphenols control the expression of microRNAs (miRNAs) in inflammation, cancer, cell differentiation, and homeostasis. Specifically, it has been demonstrated that in cancer cells, resveratrol treatments decrease the levels of several oncogenic miRNAs while increasing the levels of tumor suppressor miRNAs [16]. In 2017, an article reported changes in miRNA expression linked to the deficiency of Carnitine palmitoyltransferase-2 (CPT2), a mitochondrial enzyme involved in long-chain fatty acid entry into mitochondria for their β-oxidation and energy production. The authors demonstrated that resveratrol treatments can induce changes in miRNA expression linked to CPT2-deficiency [17].

In order to explore the role of resveratrol and its possible action mechanisms in improving female reproductive potential, we analysed miRNA profiles in Follicular Fluid (FF) samples from women of advanced reproductive age and with a poor prognosis for In Vitro Fertilization IVF, who have received Resv@MDH based supplementation for three months before undergoing IVF cycles and compared to those of women selected as the control group who had not received any supplementation. Resveratrol is poorly bioavailable because of reduced absorption mainly due to its low solubility and fast metabolism that converts it into glucuronide and sulfates compounds [18]. Solid dispersion of resveratrol supported on Magnesium DiHydroxide (Resv@MDH) has been recently developed to improve solubility and increase the bioavailability of resveratrol [18].

We decided to focus our analysis on FF because it contains different molecules produced by both the somatic and germinal components of the follicle and it has been widely demonstrated that its composition reflects the quality of the female gamete [19]. Moreover, miRNAs are able to influence the complex protein framework with fundamental roles in the numerous pathways working in the ovarian follicle and that are activated during the follicular development, oocyte maturation and acquisition competence [20]; it is also known that miRNA-altered expression can be associated with different reproductive disorders [21,22,23]. Moreover, a correlation has been reported between changes in miRNA expression and oocyte aging and epigenetics [23]. MiRNome modification in FF from aged women after resveratrol supplementation could demonstrate that the nutraceutical acts on the regulation of molecular pathways related to folliculogenesis and could improve the reproductive success in women of advanced reproductive age with a poor prognosis for IVF cycles.

## 2. Materials and Methods

### 2.1. Patients

Twelve women undergoing IVF treatment were enrolled at the Center of Reproductive Medicine and IVF Unit in Conversano, ASL Bari (Bari, Italy), between July 2019 and December 2019. All patients signed informed consent. The study protocol was approved by the local Ethical Committee (n. 5790). We evaluated the patients who satisfied the following entry criteria: women 35–42 years old with a poor ovarian reserve (AMH < 1.2 ng/mL, AFC < 5, POSEIDON group 4), excluding tumors and/or previous radio-chemotherapy, endometriosis, and severe male factors. Daily nutraceutical supplementation (trade name GENANTE^TM^) containing Resv@ MDH (total resveratrol 150 mg), folic acid (400 mcg), vitamin D (25 mcg), vitamin B12 (2.5 mcg), and vitamin B6 (1.4 mg) was used. Six women receiving this supplementation in the preceding three months of IVF cycles represent the treatment group and were compared with six women for the same period who did not receive resveratrol supplementation (ctrl, multivitamin supplementation without resveratrol). Ovarian stimulation was performed by administering Human menopausal gonadotropin (Meropur©, Ferring, Milano, Italy) at the starting dose of 300 IU–450 IU per day from the 1st or 2nd day of induced menstruation. According to the ovarian response, serial ultrasound examination and serum routine hormonal measurement (Follicular stimulating hormone, FSH; luteinizing hormone, LH; estradiol, E2, Progesterone, P) every two days, the dose of gonadotropins was adjusted. When the dominant follicle reached 14 mm in diameter, Gonadotropin-Releasing Hormone GnRH antagonist (Orgalutran©, MSD, Merck Sharp & Dohme Corp., Inc., Kenilworth, NJ, USA) was administered. Human chorionic gonadotropin (hCG) 10,000 IU s.c. (Gonasi©, IBSA, Lodi, Italy) was administered when at least two follicles reached a mean diameter of 18 mm. Oocyte retrieval was performed by a transvaginal sonography-guided technique 35–36 h after the triggering of ovulation. Basic and clinical information of all participants are presented in Table 1.

### 2.2. Follicular Fluid Sample Collection

FF samples without any flushing were collected during the aspiration of ovarian follicles (Sense™ Single lumen Needle, Vitrolife, Goteborg, Sweden), centrifugated at 2800 rpm for 20 min to remove residual follicular cells and any blood traces. The supernatant, placed into sterile polypropylene tubes, was immediately stored at −20 °C. Only FF samples with no macroscopic evidence of blood were selected.

### 2.3. RNA Isolation and Precipitation

Total RNA was extracted from 400 μL of follicular fluid samples by using Qiagen miRNeasy Mini Kit (Qiagen, GmbH, Hilden, Germany), according to Qiagen Supplementary Protocol for purification of RNA (including small RNAs) from serum or plasma. The RNA precipitation protocol was performed to increase total RNA yield. Briefly, RNA was first eluted in 200 μL of RNAse-free water and then added to 20 μg of UltraPure Glycogen (ThermoFisher), 0.1 volume of 3 M sodium acetate and 2.5 volumes of ice-cold absolute ethanol and incubated at −80 °C overnight. The day after, RNA was centrifuged and washed three times in ice-cold 75% ethanol and resuspended in 7 μL of RNAse-free water. The spectrophotometer was used to quantify total RNA before and after precipitation.

### 2.4. MiRNA Expression Profile

The expression profile of 800 miRNAs from follicular fluid samples was analyzed by the NanoString nCounter system assay through the NanoString platform and the nCounter Human v3 miRNA Expression Assay Kits (NanoString Technologies, Seattle, WA, USA), according to the manufacturer’s instructions. MiRNA expression profiling was performed on 6 FF samples from women treated with resveratrol and 6 FF samples from women not treated and used as controls. Approximately 100 ng of RNA in a final volume of 3 μL were used. Briefly, samples were processed using the automated nCounter Prep Station; after the hybridization step, they were purified and immobilized on a sample cartridge for quantification and data collection by using the nCounter Digital Analyzer. The nSolver 3.0 software was used for data analysis according to user manual instructions (https://www.nanostring.com/products/analysis-software/nsolver (accessed on 1 January 2022)).

### 2.5. MiRNA Functional Enrichment Analysis

In order to investigate the potential biological role of DE miRNAs we performed Kyoto Encyclopedia of Genes and Genomes (KEGG) pathway computational analyses by using Diana-miRPath v3.0 (http://snf-515788.vm.okeanos.grnet.gr/ (accessed on 1 January 2022)) and selected for validated mRNA targets retrieved from Tarbase7.0. The FDR method was applied to select the signaling pathways with a threshold of significance defined by *p* ≤ 0.05, a microT threshold of 0.8 and an enrichment analysis method performed by Fisher’s Exact Test (Hypergeometric Distribution). The Gene Cards Human Gene Database (https://www.genecards.org/ (accessed on 1 January 2022)) was queried to obtain two different lists of predicted and validated human genes associated with molecular signaling pathways related to the cellular response to oxidative stress and oocyte meiosis. These lists were compared to DE miRNA validated target genes retrieved by miRTarBAse (https://mirtarbase.cuhk.edu.cn/~miRTarBase/miRTarBase_2022/php/index.php (accessed on 1 January 2022)). Only target genes found in both computational analyses- the first performed in the Gene Cards Human Gene Database and the second performed in miRTarbase were selected and used to design the two regulatory networks reporting miRNA-mRNA interactions specific for cellular oxidative stress response and oocyte meiosis that were built by Cytoscape software (version 3.8.2) (https://cytoscape.org/).

### 2.6. Statistical Analysis

For miRNA profiling analysis, we performed the Volcano Plot and Significance Analysis of Microarrays (SAM) statistical tests by using MeV (Multi experiment Viewer v4.8.1) (http://mev.tm4.org/ (accessed on 1 January 2022)) software. Differentially expressed miRNAs with statistical significance were screened using the threshold log2 fold change ≥ 0.5 (log2FC) and *p*-values ≤ 0.05 corrected for multiple testing by using the Bonferroni method. SAM statistical test was performed as follows: all statistical tests were computed by applying a two-class unpaired test among log2FC and using a q-value based on 100 permutations; imputation engine: K-nearest neighbors, number of K-nearest neighbors: 10 neighbors. The Benjamini–Hochberg multiple testing correction method for high throughput analyses using a stringent false discovery rate (FDR) limit < 0.05 was applied. Unpaired *t*-test was applied for DE miRNA expression validation by using GraphPad Prism 6. Statistical significance was assessed by setting the *p*-value cut-off ≤ 0.05. Differences in IVF outcome parameters and Pearson’s correlation analyses were computed between the FC values of mitomiRNAs and biochemical pregnancy scores, by GraphPad Prism 6 (https://www.graphpad.com/ (accessed on 1 January 2022)). Statistical significance was established at *p* ≤ 0.05. Linear regression analysis was also carried out on GraphPad Prism 6 software only for tight significant correlations.

## 3. Results

### 3.1. MiRNA Expression Profiling

**Figure 1:**
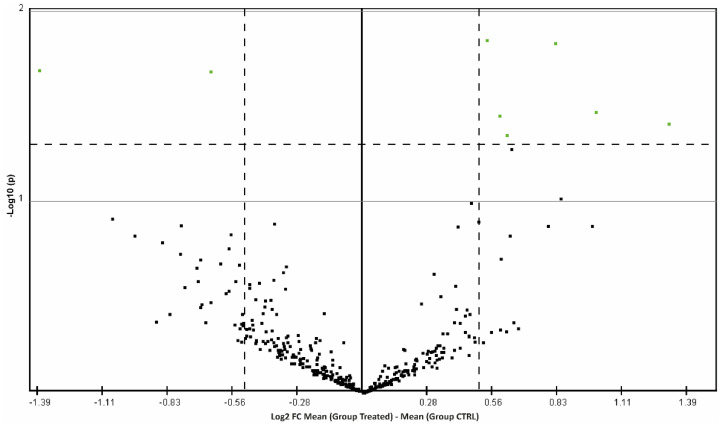
Nanostring miRNA profiling analysis. Volcano plot displaying the differences in fold change (log2FC) of miRNA expression in resveratrol treated FF samples vs. CTRL obtained after data normalization analysis. The *x*-axis indicates differences in log2FC and the *y*-axis indicates the −log10 *p*-value. The horizontal dashed line indicates the threshold for probability of significance (*p* = 0.05) and the vertical dashed lines set the threshold to 0.5 for the difference in FC of miRNA expression. miRNAs whose expression level is at least 0.5-fold different in resveratrol treated FF samples compared to CTRL, with *p* < 0.05 corrected for multiple testing by using the Bonferroni method, are indicated by green dots.

**Figure 2:**
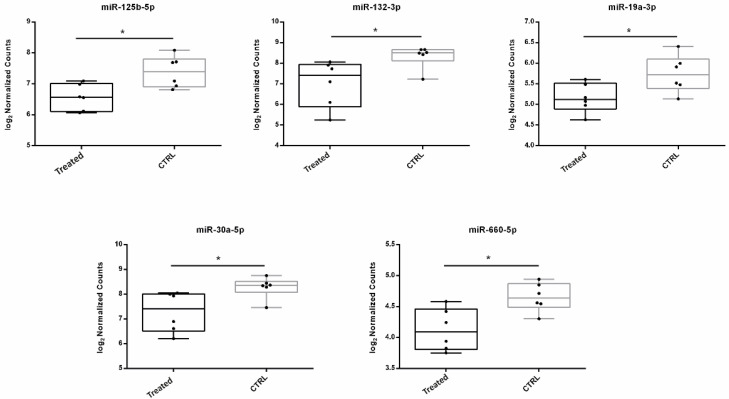
Relative expression of miRNAs in FF samples. DE miRNA relative expression in resveratrol treated FF samples vs. CTRL is shown by box-and-whisker plots. Expression data are represented as log2normalized counts. Significant *p*-values corrected for multiple testing by using the Benja-mini-Hochberg method are indicated by <<*>> (* *p*-value ≤ 0.05).

A high-throughput miRNA expression analysis of 800 miRNAs was performed in FF samples from women treated with the resveratrol-based nutraceutical supplement and women not treated by using Nanostring technology. The analysis revealed 8 differentially expressed (DE) miRNAs as shown by the volcano plot (Figure 1) and 10 DE miRNAs as revealed by SAM statistical analysis. Only DE miRNAs common to both statistical tests were chosen for further computational analyses: miR-125b-5p, miR-132-3p, miR-19a-3p, miR-30a-5p and miR-660-5p (Table 2 and Figure 2).

### 3.2. Functional Enrichment Analysis of DE miRNAs

**Figure 3:**
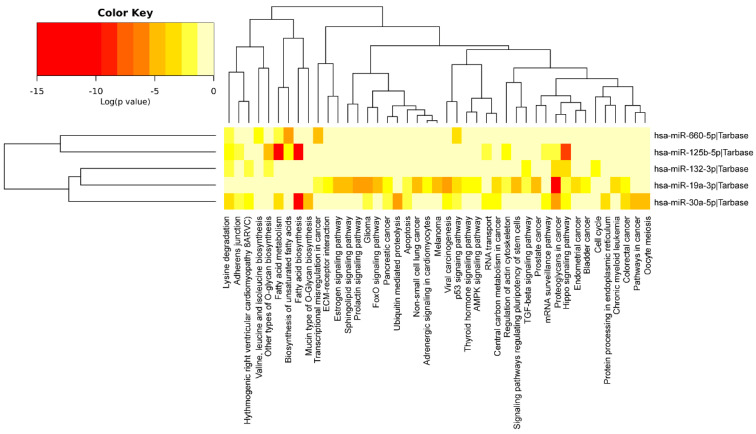
KEGG pathway analysis of DE miRNAs. Functional enrichment analysis of all DE miRNA target genes using KEGG pathway analysis. Log (*p*-value) is indicated by a yellow-red-coloured key.

**Figure 4:**
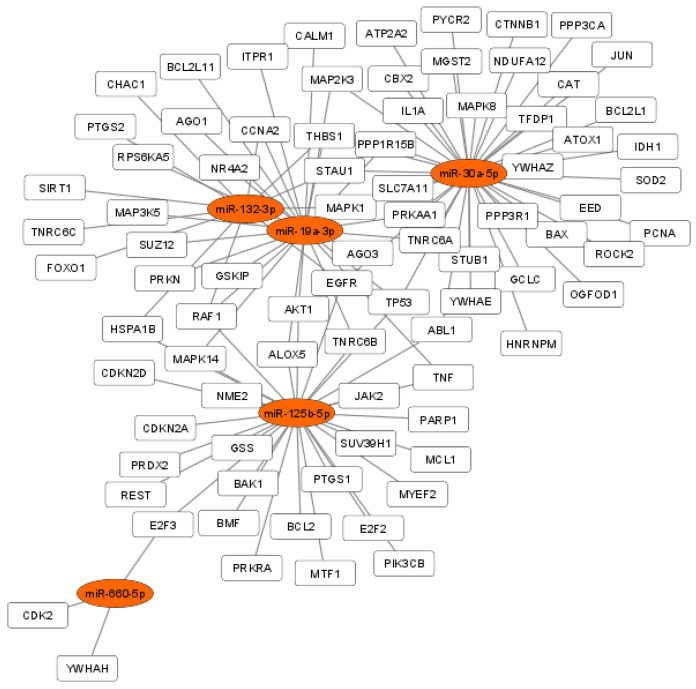
DE miRNA target genes related to the cellular response to oxidative stress. Regulatory network showing the interaction between miR-125b-5p, miR-132-3p, miR-19a-3p, miR-30a-5p and miR-660-5p and their validated mRNA targets. Orange ellipses represent miRNAs and white rectangles represent mRNA target genes involved in the cellular response to oxidative stress.

**Figure 5:**
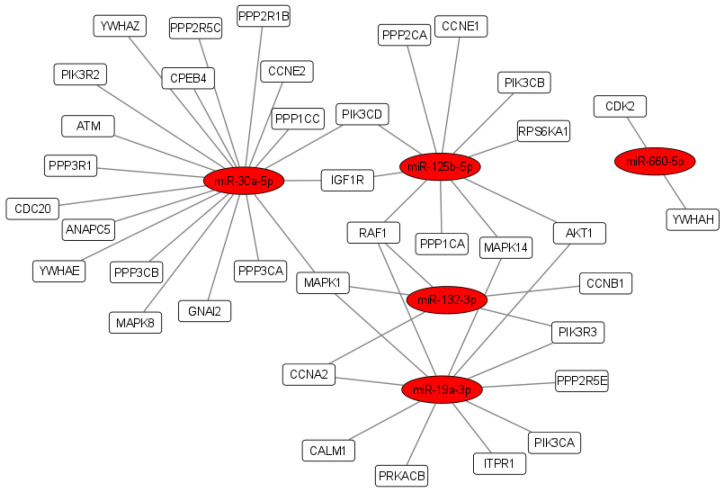
DE miRNA target genes related to oocyte meiosis. Regulatory network showing the interaction between miR-125b-5p, miR-132-3p, miR-19a-3p, miR-30a-5p, miR-660-5p and their validated mRNA targets. Red ellipses represent miRNAs and white rectangles represent mRNA target genes involved in oocyte meiosis.

We investigated DE miRNA functions for molecular signaling pathway enrichment specifically for cellular response to oxidative stress and oocyte meiosis. Functional enrichment analyses showed that the validated target genes of DE miRNAs may regulate several signaling pathways involved in fertility: fatty acid biosynthesis, estrogen signaling pathway, FOXO signaling pathway, p53 signaling pathway, TGF-beta signaling pathway, Hippo signaling pathway and oocyte meiosis (Figure 3). Regulatory network analyses showed that among the DE miRNA target genes, 84 out of 1836 were involved in the cellular response to oxidative stress (Figure 4), and 37 out of 1836 were involved in oocyte meiosis (Figure 5).

### 3.3. Impact of Resveratrol Supplementation on IVF Outcome

**Figure 6:**
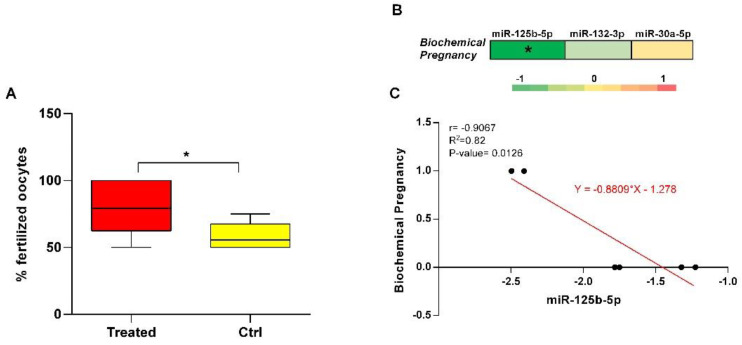
Impact of Resveratrol supplementation on IVF outcome. (**A**) Box and whisker plots showing the number (%) of fertilized good quality oocytes (MII) in treated and control groups. Statistically significant *p*-values (*p* ≤ 0.05) are indicated by asterisks. (**B**) Correlation matrix obtained by calculating Pearson correlation coefficients for mitomiR expression (FC) and biochemical pregnancy scores. The correlation values are indicated by a color gradient from green (negative correlation) to red (positive correlation), as shown in the colored bar. Statistically significant *p*-values (*p* ≤ 0.05) are indicated by asterisks. (**C**) Scatterplot on miRNAs tightly correlated and showing the best-fit line obtained from linear regression analysis.

The mean number of fertilized good quality oocytes when patients received supplementation was significantly increased from 63% to 80% (Figure 6A). By comparing treated and control patients, we found a significant negative relationship between the biochemical pregnancy scores and miR-125b-5p (*r* = −0.91), a lower negative relationship between the biochemical pregnancy scores and miR-132-3p (*r* = −0.43), whereas there was no correlation between biochemical pregnancy scores and miR-30a-5p (*r* = 0.05) (Figure 6B,C).

## 4. Discussion

Over the years, a women’s ovarian reserve, made up during intrauterine life, is gradually depleted and the quality of oocytes becomes lower for the increase in chromosomal aneuploidies, decreasing mitochondrial quality and impaired balance between oxidative stress and antioxidant defenses [24]. Female reproductive aging reduces fertility and pregnancy outcomes and represents a critical problem in developed countries [25]. Considering that women often have to delay their first pregnancy for professional reasons and Assisted Reproductive Technology (ART) is frequently inefficacious in ovarian ageing, it is becoming increasingly important to design innovative strategies to improve pregnancy success in women over 35 years of age. Resveratrol is a nutraceutical with several therapeutic effects. It has been shown to exert anti-inflammatory and anti-oxidative effects and affect the initiation and progression of many diseases through different mechanisms [26]. Several studies seem to demonstrate that resveratrol improves male and female reproductive function even if the mechanisms of action and the therapeutic effects remain not fully clarified [27]. Recently, in an in vitro model, Ragonese and collaborators demonstrated that resveratrol improved ATP production and cell viability and promoted the induction of cellular differentiation, increasing mitochondrial biogenesis, in granulosa cells [14]. To evaluate the biological effects of the resveratrol nutraceutical in vivo, we investigated miRNA profiles in FF, comparing their expression between aged women treated with resveratrol and untreated controls. We found that the treatment induces important variations of miRNA content in FF and specifically the significant downregulation of miR-19a-3p, miR-30a-5p miR-125b-5p, miR-132-3p and miR-660-5p (Figure 1 and Table 2). It is known that miRNAs can influence mitochondrial activity in different ways. Nuclear miRNAs can regulate mitochondrial proteins encoded by the nuclear genome alternatively, after their translocation inside the mitochondrial matrix, proteins encoded by mitochondrial DNA [28]. MiRNAs could also be transcribed from the mitochondrial genome and after maturation inside the mitochondria, the miRNAs could inhibit mRNA translation in the mitochondrial and cytosolic compartments. There is still little experimental evidence of miRNAs encoded by the mitochondrial genome, instead, to date, about 150 nuclear miRNAs have been detected in mitochondria of different species (MitomiRs). MitomiRs are encoded by the nuclear genome, imported into mitochondria and involved in mitochondrial and nuclear genome regulation. They can regulate metabolism and mitochondrial biogenesis even if, the details of how they are imported into the mitochondria or how they regulate mitochondrial gene expression need to be addressed [29]. Notwithstanding, the role of mitomiRs has been widely demonstrated in the pathogenesis of cardiovascular diseases and neurodegenerative pathologies [29]. Three of the identified downregulated miRNAs, miR-30a-5p miR-125b-5p, miR-132-3p are mitomiRs. MiR-30a-5p can regulate mitochondria fission and apoptosis through TP53 and Dynamin-related protein 1 (DRP1). DRP1, interacting with other proteins, as mitochondrial fission factor (MFF), induces the mitochondrial division and its transcription is promoted by TP53. TP53 is a validated target of miR-30a-5p, accordingly, the downregulation of miR-30a-5p could indirectly upregulate DRP1 enhancing mitochondrion fission [30].

Carnitine-acyl carnitine translocase (CACT) is a critical mitochondrial carrier involved in lipid metabolism. CACT catalyzes both unidirectional transport of carnitine and carnitine/acylcarnitine exchange in the inner mitochondrial membrane, allowing the import of long-chain fatty acids into the mitochondria where they are oxidized by the β-oxidation pathway. Its inactivation, mediated by the upregulation of miR-132, has been demonstrated in obese mice; consequently, the downregulation of miR-132, after resveratrol treatment, could increase fatty acid catabolism which is an important source of energy for the cells [31]. In monocytes, miR-125b-5p reduces mitochondrial respiration through BIK silencing and plays an important role in the repression of Brite adipocyte function by modulating oxygen consumption and mitochondrial gene expression [32].

We propose that miR-30a-5p, miR-132 and miR-125b-5p downregulation, induced by resveratrol treatment in aged women, could improve oocyte quality by enhancing mitochondrial activity and increasing ATP production in granulosa cells. Interestingly, we found that the number of fertilized good quality oocytes increases in treated women and a significant anticorrelation between miR-125-fold change values and biochemical pregnancy is present (Figure 6). The effect of resveratrol in improving reproductive potential has already been demonstrated both in vitro and in vivo; in fact, it increases mitochondrial activity and biogenesis, in granulosa cell cultures [14] and clinically improves folliculogenesis outcome during IntraCytoplasmic Sperm Injection (ICSI) cycles [33].

Our data suggest that resveratrol treatment induces modification in the granulose cell miRNome suggesting that the different effects of nutriceutical could also depend on the regulation of gene expression mediated by miRNAs. Moreover, different papers reported that resveratrol can modulate miRNA expression in vitro, and among the miRNAs downregulated, miR-125b-5p has been described [34,35]. Of course, miRNAs perform their biological function inside the cells, and their altered expression in biological fluids does not always match their intracellular profile. MiRNA expression levels in biological fluids may be due to both changes in the transcription levels inside the cells producing them and alteration of secretion mechanisms [23,36]. In any case, the expression differences observed in FF after resveratrol treatment surely reflect transcriptomic and proteomic modification in granulose cells.

Functional enrichment analysis on downregulated miRNAs revealed that they may regulate several signaling pathways involved in mitochondria function and female fertility (Figure 2). Fatty acid biosynthesis, estrogen, FOXO, p53, TGF-beta, Hippo signaling pathways and oocyte meiosis represent some of the signaling pathways regulated by the five miRNAs (Figure 3). Interestingly, some literature data confirm these analyses. Zhang et al. investigated steroid hormone concentrations and miR-125b-5p expression in PCOS mouse model. In preantral follicles, inhibition of miR-125b-5p increased the expression of androgen synthesis related-genes and stimulated the secretion of testosterone, while simultaneously downregulating oestrogen synthesis-related genes and decreasing oestradiol release [37]. It is well known that androgens can influence ovarian follicular growth, augment steroidogenesis, promote follicular recruitment and increase the number of primary and preantral follicles [38]. Upregulation of miR-19a-3p has been found associated with an increase in inflammation [39] and its inhibition would seem to mitigate the repression of glycolysis enzymes, glucose uptake and lactate production, and apoptosis induced by an ischemic stroke in neurons [40].

MiR-125b-5p, miR-132-3p, miR-19a-3p, miR-30a-5p and miR-660-5p act synergistically in the networks related to the cellular response to oxidative stress and oocyte maturation and some of their target genes as SIRT1, TNF-a, AKT, have been previously identified as potential effectors of the cellular response to the resveratrol (Figure 3 and Figure 4) [41,42]. SIRT1, acting at different regulation levels, activates the mechanisms of oxidative stress response in human reproductive organs. In the female reproductive system, SIRT1 regulates proliferation and apoptosis in granulosa cells (GCs), and its downregulation is associated with a reduced ovarian reserve [43]. The decrease in its expression causes mitochondrial dysfunction by increasing the Reactive Oxygen Species (ROS), lipid peroxidation and DNA damage in oocytes. In women of advanced reproductive age, SIRT1 by the deacetylation of the FOXO3A transcription factor stimulates the expression of catalase and manganese superoxide dismutase, promoting cell survival under oxidant conditions [44,45]. MiR-132 has SIRT1 as a validated target and its downregulation in granulosa cells could be associated with SIRT1 upregulation, and represent a response, mediated by resveratrol, to oxidative stress.

## 5. Conclusions

We propose that resveratrol treatment, by inducing modification in the granulose cell miRNome acting specifically on the miRNAs involved in mitochondrial pathways, improves oocyte quality and pregnancy outcome. A better understanding of the roles of nutriceutical on mitochondrial miRNAs will open up the possibility of designing new therapeutic strategies for mitochondrial diseases to improve follicular microenvironment in women of advanced reproductive age increasing pregnancy success in natural and IVF cycles.

## Figures and Tables

**Table 1:** Clinical parameters of women enrolled in the study. Values are reported as mean ± standard deviation or percentage (%). *p*-values are based on a two-sample *t*-test. MBI: body mass index; AMH: anti-Müllerian hormone.

Parameters	Study Group	Control Group	*p*-Value
Patients (N°)	6	6	
Age (Years)	38 ± 3.3	39 ± 3	0.29
BMI (kg/m^2^)	23.6 ± 3.15	22.16 ± 1.8	0.37
AMH (ng/mL)	0.72 ± 0.32	0.77 ± 0.43	0.31
Antral Follicle Count (N°)	3.83 ± 1	4.16 ± 1	0.29
Gonadotropin dosage (IU)	3425 ± 799	3750 ± 1152	0.28
Stimulation protocol length (days)	10.8 ± 1.2	10.8 ± 1.2	0.5
Follicles (N°)	8.6 ± 6.5	7 ± 4	0.29
MII oocytes (N°)	5.5 ± 3.7	5.8 ± 2.7	0.4
Pregnancy rate (%)	50%	33%	

**Table 2:** List of DE miRNAs in resveratrol treated FF samples vs. CTRL. DE miRNAs were selected according to Volcano Plot and SAM statistical tests. DE miRNAs common to both statistical tests and chosen for further analyses are highlighted in bold. The FC value of each miRNA is reported.

DE miRNAs	Ttest	SAM	Fold Change Treated vs. Ctrl
miR-1180-3p		X	−1.55
miR-125b-5p	**X**	**X**	−1.76
miR-132-3p	**X**	**X**	−2.47
miR-16-5p		X	−1.73
miR-195-5p	X		−1.53
miR-19a-3p	**X**	**X**	−1.5
miR-30a-5p	**X**	**X**	−1.99
miR-30d-5p		X	−1.97
miR-323a-3p	X		1.57
miR-365a-3p + miR-365b-3p		X	−1.79
miR-497-5p		X	−1.41
miR-574-5p	X		2.62
miR-660-5p	**X**	**X**	−1.44

## Data Availability

The data presented in this study are available in this manuscript.

## References

[1] Yureneva S., Averkova V., Silachev D., Donnikov A., Gavisova A., Serov V., Sukhikh G. (2021). Searching for female reproductive aging and longevity biomarkers. Aging.

[2] Deatsman S., Vasilopoulos T., Rhoton-Vlasak A. (2016). Age and Fertility: A Study on Patient Awareness. JBRA Assist. Reprod..

[3] Ruth K.S., Day F.R., Hussain J., Martinez-Marchal A., Aiken C.E., Azad A., Thompson D.J., Knoblochova L., Abe H., Tarry-Adkins J.L. (2021). Genetic insights into biological mechanisms governing human ovarian ageing. Nature.

[4] Santonocito M., Guglielmino M.R., Vento M., Ragusa M., Barbagallo D., Borzi P., Casciano I., Scollo P., Romani M., Tatone C. (2013). The apoptotic transcriptome of the human MII oocyte: Characterization and age-related changes. Apoptosis.

[5] Webster A., Schuh M. (2017). Mechanisms of Aneuploidy in Human Eggs. Trends Cell Biol..

[6] Zhang D., Keilty D., Zhang Z.F., Chian R.C. (2017). Mitochondria in oocyte aging: Current understanding. Facts Views Vis. ObGyn.

[7] Eichenlaub-Ritter U. (2012). Oocyte ageing and its cellular basis. Int. J. Dev. Biol..

[8] Luddi A., Capaldo A., Focarelli R., Gori M., Morgante G., Piomboni P., De Leo V. (2016). Antioxidants reduce oxidative stress in follicular fluid of aged women undergoing IVF. Reprod. Biol. Endocrinol..

[9] Posadino A.M., Cossu A., Giordo R., Zinellu A., Sotgia S., Vardeu A., Hoa P.T., Van Nguyen L.H., Carru C., Pintus G. (2015). Resveratrol alters human endothelial cells redox state and causes mitochondrial-dependent cell death. Food Chem. Toxicol..

[10] Shaito A., Posadino A.M., Younes N., Hasan H., Halabi S., Alhababi D., Al-Mohannadi A., Abdel-Rahman W.M., Eid A.H., Nasrallah G.K. (2020). Potential Adverse Effects of Resveratrol: A Literature Review. Int. J. Mol. Sci..

[11] Plauth A., Geikowski A., Cichon S., Wowro S.J., Liedgens L., Rousseau M., Weidner C., Fuhr L., Kliem M., Jenkins G. (2016). Hormetic shifting of redox environment by pro-oxidative resveratrol protects cells against stress. Free Radic. Biol. Med..

[12] Lagouge M., Argmann C., Gerhart-Hines Z., Meziane H., Lerin C., Daussin F., Messadeq N., Milne J., Lambert P., Elliott P. (2006). Resveratrol improves mitochondrial function and protects against metabolic disease by activating SIRT1 and PGC-1alpha. Cell.

[13] Tatone C., Di Emidio G., Barbonetti A., Carta G., Luciano A.M., Falone S., Amicarelli F. (2018). Sirtuins in gamete biology and reproductive physiology: Emerging roles and therapeutic potential in female and male infertility. Hum. Reprod. Update.

[14] Ragonese F., Monarca L., De Luca A., Mancinelli L., Mariani M., Corbucci C., Gerli S., Iannitti R.G., Leonardi L., Fioretti B. (2021). Resveratrol depolarizes the membrane potential in human granulosa cells and promotes mitochondrial biogenesis. Fertil. Steril..

[15] Rodriguez-Varela C., Labarta E. (2020). Clinical Application of Antioxidants to Improve Human Oocyte Mitochondrial Function: A Review. Antioxidants.

[16] Latruffe N., Lancon A., Frazzi R., Aires V., Delmas D., Michaille J.J., Djouadi F., Bastin J., Cherkaoui-Malki M. (2015). Exploring new ways of regulation by resveratrol involving miRNAs, with emphasis on inflammation. Ann. N. Y. Acad. Sci..

[17] Aires V., Delmas D., Djouadi F., Bastin J., Cherkaoui-Malki M., Latruffe N. (2017). Resveratrol-Induced Changes in MicroRNA Expression in Primary Human Fibroblasts Harboring Carnitine-Palmitoyl Transferase-2 Gene Mutation, Leading to Fatty Acid Oxidation Deficiency. Molecules.

[18] Spogli R., Bastianini M., Ragonese F., Iannitti R.G., Monarca L., Bastioli F., Nakashidze I., Brecchia G., Menchetti L., Codini M. (2018). Solid Dispersion of Resveratrol Supported on Magnesium DiHydroxide (Resv@MDH) Microparticles Improves Oral Bioavailability. Nutrients.

[19] Di Pietro C. (2016). Exosome-mediated communication in the ovarian follicle. J. Assist. Reprod. Genet..

[20] Bianchi L., Gagliardi A., Landi C., Focarelli R., De Leo V., Luddi A., Bini L., Piomboni P. (2016). Protein pathways working in human follicular fluid: The future for tailored IVF?. Expert Rev. Mol. Med..

[21] Di Pietro C., Caruso S., Battaglia R., Iraci Sareri M., La Ferlita A., Strino F., Bonaventura G., Di Mauro M., Barcellona M.L., Perciavalle V. (2018). MiR-27a-3p and miR-124-3p, upregulated in endometrium and serum from women affected by Chronic Endometritis, are new potential molecular markers of endometrial receptivity. Am. J. Reprod. Immunol..

[22] Di Pietro C., Vento M., Guglielmino M.R., Borzi P., Santonocito M., Ragusa M., Barbagallo D., Duro L.R., Majorana A., De Palma A. (2010). Molecular profiling of human oocytes after vitrification strongly suggests that they are biologically comparable with freshly isolated gametes. Fertil. Steril..

[23] Battaglia R., Vento M.E., Ragusa M., Barbagallo D., La Ferlita A., Di Emidio G., Borzi P., Artini P.G., Scollo P., Tatone C. (2016). MicroRNAs Are Stored in Human MII Oocyte and Their Expression Profile Changes in Reproductive. Aging Biol. Reprod..

[24] May-Panloup P., Boucret L., Chao de la Barca J.M., Desquiret-Dumas V., Ferre-L’Hotellier V., Moriniere C., Descamps P., Procaccio V., Reynier P. (2016). Ovarian ageing: The role of mitochondria in oocytes and follicles. Hum. Reprod. Update.

[25] Nelson S.M., Telfer E.E., Anderson R.A. (2013). The ageing ovary and uterus: New biological insights. Hum. Reprod. Update.

[26] Berman A.Y., Motechin R.A., Wiesenfeld M.Y., Holz M.K. (2017). The therapeutic potential of resveratrol: A review of clinical trials. NPJ Precis. Oncol..

[27] Pasquariello R., Verdile N., Brevini T.A.L., Gandolfi F., Boiti C., Zerani M., Maranesi M. (2020). The Role of Resveratrol in Mammalian Reproduction. Molecules.

[28] Geiger J., Dalgaard L.T. (2017). Interplay of mitochondrial metabolism and microRNAs. Cell. Mol. Life Sci..

[29] John A., Kubosumi A., Reddy P.H. (2020). Mitochondrial MicroRNAs in Aging and Neurodegenerative Diseases. Cells.

[30] Li J., Donath S., Li Y., Qin D., Prabhakar B.S., Li P. (2010). miR-30 regulates mitochondrial fission through targeting p53 and the dynamin-related protein-1 pathway. PLoS Genet..

[31] Soni M.S., Rabaglia M.E., Bhatnagar S., Shang J., Ilkayeva O., Mynatt R., Zhou Y.P., Schadt E.E., Thornberry N.A., Muoio D.M. (2014). Downregulation of carnitine acyl-carnitine translocase by miRNAs 132 and 212 amplifies glucose-stimulated insulin secretion. Diabetes.

[32] Giroud M., Pisani D.F., Karbiener M., Barquissau V., Ghandour R.A., Tews D., Fischer-Posovszky P., Chambard J.C., Knippschild U., Niemi T. (2016). miR-125b affects mitochondrial biogenesis and impairs brite adipocyte formation and function. Mol. Metab..

[33] Gerli S., Della Morte C., Ceccobelli M., Mariani M., Favilli A., Leonardi L., Lanti A., Iannitti R.G., Fioretti B. (2021). Biological and clinical effects of a resveratrol-based multivitamin supplement on intracytoplasmic sperm injection cycles: A single-center, randomized controlled trial. J. Matern. Fetal Neonatal Med..

[34] Vallino L., Ferraresi A., Vidoni C., Secomandi E., Esposito A., Dhanasekaran D.N., Isidoro C. (2020). Modulation of non-coding RNAs by resveratrol in ovarian cancer cells: In silico analysis and literature review of the anti-cancer pathways involved. J. Tradit. Complement. Med..

[35] Venkatadri R., Muni T., Iyer A.K., Yakisich J.S., Azad N. (2016). Role of apoptosis-related miRNAs in resveratrol-induced breast cancer cell death. Cell Death Dis..

[36] Gebremedhn S., Ali A., Gad A., Prochazka R., Tesfaye D. (2020). Extracellular Vesicles as Mediators of Environmental and Metabolic Stress Coping Mechanisms during Mammalian Follicular Development. Front. Vet. Sci..

[37] Zhang X., Xiao H., Zhang X., Qiukai E., Gong X., Li T., Han Y., Ying X., Cherrington B.D., Xu B. (2020). Decreased microRNA-125b-5p disrupts follicle steroidogenesis through targeting PAK3/ERK1/2 signalling in mouse preantral follicles. Metabolism.

[38] Noventa M., Vitagliano A., Andrisani A., Blaganje M., Vigano P., Papaelo E., Scioscia M., Cavallin F., Ambrosini G., Cozzolino M. (2019). Testosterone therapy for women with poor ovarian response undergoing IVF: A meta-analysis of randomized controlled trials. J. Assist. Reprod. Genet..

[39] Ye L., Morse L.R., Falci S.P., Olson J.K., Shrivastava M., Nguyen N., Linnman C., Troy K.L., Battaglino R.A. (2021). hsa-MiR-19a-3p and hsa-MiR-19b-3p Are Associated with Spinal Cord Injury-Induced Neuropathic Pain: Findings from a Genome-Wide MicroRNA Expression Profiling Screen. Neurotrauma Rep..

[40] Ge X.L., Wang J.L., Liu X., Zhang J., Liu C., Guo L. (2019). Inhibition of miR-19a protects neurons against ischemic stroke through modulating glucose metabolism and neuronal apoptosis. Cell. Mol. Biol. Lett..

[41] McCubrey J.A., Lertpiriyapong K., Steelman L.S., Abrams S.L., Yang L.V., Murata R.M., Rosalen P.L., Scalisi A., Neri L.M., Cocco L. (2017). Effects of resveratrol, curcumin, berberine and other nutraceuticals on aging, cancer development, cancer stem cells and microRNAs. Aging.

[42] Artini P.G., Tatone C., Sperduti S., D’Aurora M., Franchi S., Di Emidio G., Ciriminna R., Vento M., Di Pietro C., Stuppia L. (2017). Cumulus cells surrounding oocytes with high developmental competence exhibit down-regulation of phosphoinositol 1,3 kinase/protein kinase B (PI3K/AKT) signalling genes involved in proliferation and survival. Hum. Reprod..

[43] Tatone C., Di Emidio G., Vitti M., Di Carlo M., Santini S., D’Alessandro A.M., Falone S., Amicarelli F. (2015). Sirtuin Functions in Female Fertility: Possible Role in Oxidative Stress and Aging. Oxidative Med. Cell. Longev..

[44] Zhou Y., Li K.S., Liu L., Li S.L. (2020). MicroRNA132 promotes oxidative stressinduced pyroptosis by targeting sirtuin 1 in myocardial ischaemiareperfusion injury. Int. J. Mol. Med..

[45] Di Emidio G., Falone S., Vitti M., D’Alessandro A.M., Vento M., Di Pietro C., Amicarelli F., Tatone C. (2014). SIRT1 signalling protects mouse oocytes against oxidative stress and is deregulated during aging. Hum. Reprod..

